# 3-{[(Di­benzyl­carbamo­thio­yl)amino]­carbon­yl}benzamide

**DOI:** 10.1107/S1600536813017467

**Published:** 2013-06-29

**Authors:** N. Selvakumaran, R. Karvembu, Seik Weng Ng, Edward R. T. Tiekink

**Affiliations:** a Department of Chemistry, National Institute of Technology, Tiruchirappalli 620 015, India; b Department of Chemistry, University of Malaya, 50603 Kuala Lumpur, Malaysia; c Chemistry Department, Faculty of Science, King Abdulaziz University, PO Box 80203 Jeddah, Saudi Arabia

## Abstract

Two independent mol­ecules with quite similar conformations, *A* and *B*, comprise the asymmetric unit of the title compound, C_23_H_21_N_3_O_2_S. The terminal amide substituent is coplanar with the attached benzene ring [the O—C—C—C torsion angles are 174.0 (2) (*A*) and 6.3 (3)° *(B*)]. In the same way, the central amide group [C—C—C—O = 7.8 (3) (*A*) and 11.5 (3)° (*B*)] is approximately coplanar with the ring to which it is attached. A major twist is noted between the amide and adjacent thio­amide residues [C—N—C—S = −109.29 (19) *(A*) and −112.29 (19)° *(B*)]. In the crystal, supra­molecular chains along [100] are formed by N—H⋯O and N—H⋯S hydrogen bonding. These are connected into a three-dimensional architecture by C—H⋯π and π–π inter­actions [inter-centroid distance = 3.9157 (12) Å].

## Related literature

For the preparation of bipodal acyl­thio­urea derivatives, see: Bourne *et al.* (2005 ▶). For a related structure, see: Selvakumaran *et al.* (2013 ▶).
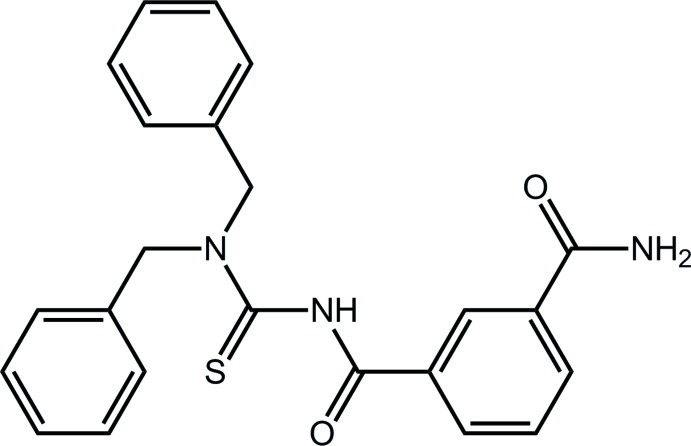


## Experimental

### 

#### Crystal data

C_23_H_21_N_3_O_2_S
*M* *_r_* = 403.49
Monoclinic, 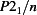

*a* = 11.3448 (5) Å
*b* = 18.6100 (8) Å
*c* = 19.3282 (7) Å
β = 97.297 (4)°
*V* = 4047.7 (3) Å^3^
*Z* = 8
Mo *K*α radiation
μ = 0.18 mm^−1^
*T* = 100 K
0.40 × 0.40 × 0.40 mm

#### Data collection

Agilent SuperNova Dual diffractometer with an Atlas detector
Absorption correction: multi-scan (*CrysAlis PRO*; Agilent, 2013 ▶) *T*_min_ = 0.827, *T*_max_ = 1.000
41058 measured reflections
9360 independent reflections
6863 reflections with *I* > 2σ(*I*)
*R*_int_ = 0.067

#### Refinement

*R*[*F* ^2^ > 2σ(*F* ^2^)] = 0.058
*wR*(*F* ^2^) = 0.124
*S* = 1.03
9360 reflections
541 parameters
6 restraints
H atoms treated by a mixture of independent and constrained refinement
Δρ_max_ = 0.32 e Å^−3^
Δρ_min_ = −0.29 e Å^−3^

### 

Data collection: *CrysAlis PRO* (Agilent, 2013 ▶); cell refinement: *CrysAlis PRO*; data reduction: *CrysAlis PRO*; program(s) used to solve structure: *SHELXS97* (Sheldrick, 2008 ▶); program(s) used to refine structure: *SHELXL97* (Sheldrick, 2008 ▶); molecular graphics: *ORTEP-3 for Windows* (Farrugia, 2012 ▶), QMol (Gans & Shalloway, 2001 ▶) and *DIAMOND* (Brandenburg, 2006 ▶); software used to prepare material for publication: *publCIF* (Westrip, 2010 ▶).

## Supplementary Material

Crystal structure: contains datablock(s) global, I. DOI: 10.1107/S1600536813017467/hg5327sup1.cif

Structure factors: contains datablock(s) I. DOI: 10.1107/S1600536813017467/hg5327Isup2.hkl

Supplementary material file. DOI: 10.1107/S1600536813017467/hg5327Isup3.cml

Additional supplementary materials:  crystallographic information; 3D view; checkCIF report

## Figures and Tables

**Table 1:** Hydrogen-bond geometry (Å, °) *Cg*1–*Cg*3 are the centroids of the C25–C30, C34–C39 and C11–C16 benzene rings, respectively.

*D*—H⋯*A*	*D*—H	H⋯*A*	*D*⋯*A*	*D*—H⋯*A*
N1—H12⋯S1^i^	0.88 (1)	2.60 (1)	3.4103 (19)	155 (2)
N1—H11⋯S2	0.88 (1)	2.65 (1)	3.5220 (19)	170 (2)
N2—H2⋯O3	0.87 (1)	2.01 (1)	2.832 (2)	156 (2)
N4—H41⋯S1	0.88 (1)	2.61 (1)	3.4666 (19)	165 (2)
N4—H42⋯S2^ii^	0.88 (1)	2.65 (2)	3.437 (2)	150 (2)
N5—H5⋯O1	0.87 (1)	2.09 (1)	2.909 (2)	156 (2)
C13—H13⋯*Cg*1^iii^	0.95	2.92	3.614 (3)	130
C17—H17*A*⋯*Cg*2^iii^	0.99	2.90	3.644 (2)	132
C40—H40*A*⋯*Cg*3^iv^	0.99	2.86	3.549 (2)	128

Symmetry codes: (i) 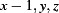; (ii) 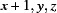; (iii) 
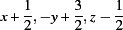
; (iv) 
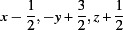
.

**Fig. 1.**
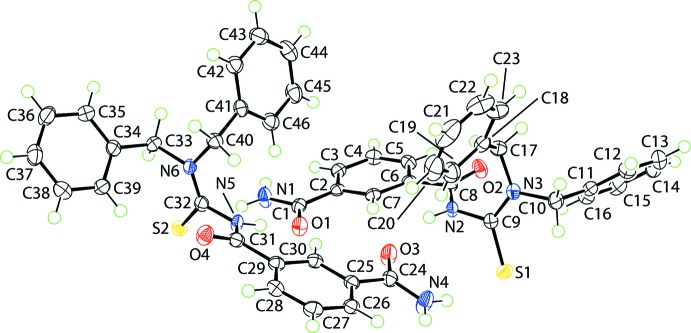
Molecular structures of the two independent molecules comprising the asymmetric unit of (I) showing atom-labelling scheme and displacement ellipsoids at the 70% probability level.

**Fig. 2.**
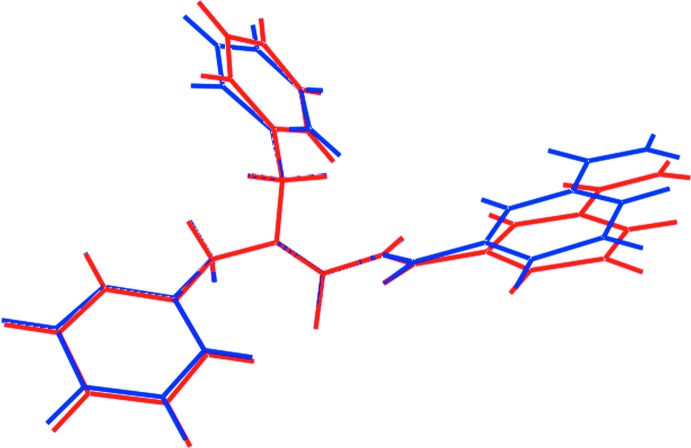
Overlay diagram of the two independent molecules in (I). The molecules have been superimposed so that the N—C—N atoms are overlapped. Red image: S1-containing molecule. Blue image, S2-containing molecule.

**Fig. 3.**
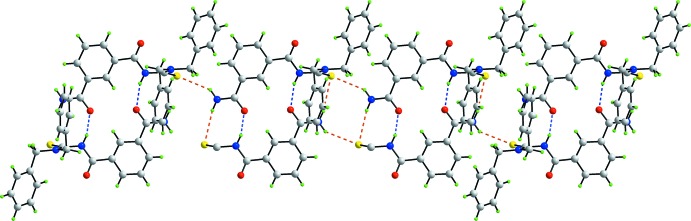
A view of the supramolecular chain along the a axis sustained by N—H···O and N—H···S hydrogen bonding shown as blue and orange dashed lies, respectively.

**Fig. 4.**
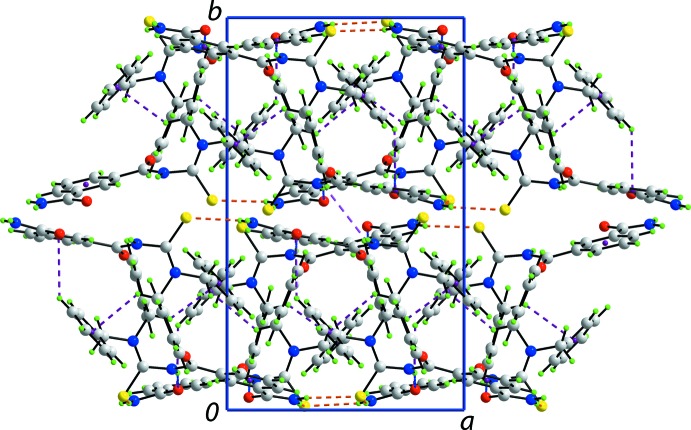
A view of the unit-cell contents in projection down the c axis in (I). The N—H···O, N—H···S, C—H···π and π—π interactions are shown as blue, orange, purple and pink dashed lines, respectively.

## supplementary crystallographic information

### Comment

In an attempt to prepare a bipodal acylthiourea derivative (Bourne *et al.*, 2005) from dibenzylamine, isophthaloyl dichloride and potassium thiocyanate in acetone, the title compound, (I), was obtained as a by-product.

Two independent but similar molecules comprise the asymmetric unit in (I), Fig. 1. In each case, the terminal amide substituent is co-planar with the attached benzene ring as seen in the O1—C1—C2—C3 and O3—C24—C25—C30 torsion angles of 174.0 (2) and 6.3 (3)°, respectively. Similarly, the central amide [C5—C6—C8—O2 = 7.8 (3)° and C28—C29—C31—O4 = 11.5 (3)°] is approximately co-planar with the ring to which it is attached. It is in this region of the molecule that the major differences occur although minimal, Fig. 2. A major twist is noted between the amide and adjacent thioamide residues as seen in the torsion angles: C8—N2—C9—S1 = -109.29 (19)° and C31—N5—C32—S2 = -112.29 (19)°. The benzyl substituents lie to either side and are approximately perpendicular to the C_3_N plane with the C9—N3—C10—C11 and C9—N3—C17–C18 torsion angles being 98.2 (2) and 95.8 (2)°, respectively, for the first independent molecule. For the second independent molecule, the C32—N6—C33—C34 torsion angle is 100.5 (2)° and the C32—N6—C40—C41 angle is 96.9 (2)°. To a first approximation, the observed conformation matches that reported in the accompanying paper (Selvakumaran *et al.*, 2013).

In the crystal packing, supramolecular chains along the *a* axis are formed by N—O and N—H···S hydrogen bonds, Fig. 3 and Table 1. These are connected into layers by C—H···π interactions, Table 1. A three-dimensional architecture is formed *via*π—π interactions between centrosymmetrically related benzene rings [inter-centroid distance = 3.9157 (12) Å for symmetry operation 1*-x*, 1 - *y*, 1 - *z*], Fig. 4.

### Experimental

Isophthaloyl dichloride (2.0302 g, 10 mmol) dissolved in acetone (80 ml), was placed in a dropping funnel and added drop wise with stirring to potassium thiocyanate (1.9436 g, 20 mmol) dissolved in acetone (80 ml), under N_2_ atmosphere, in a three-necked round bottom flask. The mixture was heated to reflux for 30 minutes and then allowed to cool. A solution of dibenzylamine (3.9456 g, 20 mmol) in acetone (80 ml) was added drop wise from a dropping funnel to the reaction mixture and the resulting mixture was stirred for 2 h at room temperature. Then, hydrochloric acid (0.1 N, 300 ml) was added and the resulting white solid was filtered off, washed with water and dried *in vacuo*. Single crystals were grown at room temperature from acetonitrile/dimethyl formamide mixture (1:1). FT—IR (KBr): ν(NH_2_) 3290 & 3225, ν(N—H) 3426, ν(C═O) 1674 (adjacent to NH_2_),ν(C═O) 1660 (adjacent to NH), ν(C═C) 1599, ν(C═S) 1257 cm^-1^. UV-Vis (DMF): λ_max_; 267, 281, 359 nm.

### Refinement

Carbon-bound H-atoms were placed in calculated positions [C—H = 0.95 to 0.98 Å, *U*_iso_(H)= 1.2*U*_eq_(C)] and were included in the refinement in the riding model approximation. The N-bound H-atoms were refined with N—H = 0.88±0.01 Å, and with *U*_iso_(H)= 1.2*U*_eq_(N). A reflection, *i.e*. (0 1 2), was omitted owing to poor agreement.

### Crystal data

**Table:**

C_23_H_21_N_3_O_2_S	*F*(000) = 1696
*M**_r_* = 403.49	*D*_x_ = 1.324 Mg m^−^^3^
Monoclinic, *P*2_1_/*n*	Mo *K*α radiation, λ = 0.71073 Å
Hall symbol: -P 2yn	Cell parameters from 8114 reflections
*a* = 11.3448 (5) Å	θ = 2.3–27.5°
*b* = 18.6100 (8) Å	µ = 0.18 mm^−^^1^
*c* = 19.3282 (7) Å	*T* = 100 K
β = 97.297 (4)°	Block, colourless
*V* = 4047.7 (3) Å^3^	0.40 × 0.40 × 0.40 mm
*Z* = 8	

### Data collection

**Table:**

Agilent SuperNova Dual diffractometer with an Atlas detector	9360 independent reflections
Radiation source: SuperNova (Mo) X-ray Source	6863 reflections with *I* > 2σ(*I*)
Mirror monochromator	*R*_int_ = 0.067
Detector resolution: 10.4041 pixels mm^-1^	θ_max_ = 27.6°, θ_min_ = 2.3°
ω scan	*h* = −12→14
Absorption correction: multi-scan (*CrysAlis PRO*; Agilent, 2013)	*k* = −24→24
*T*_min_ = 0.827, *T*_max_ = 1.000	*l* = −25→25
41058 measured reflections	

### Refinement

**Table:**

Refinement on *F*^2^	Primary atom site location: structure-invariant direct methods
Least-squares matrix: full	Secondary atom site location: difference Fourier map
*R*[*F*^2^ > 2σ(*F*^2^)] = 0.058	Hydrogen site location: inferred from neighbouring sites
*wR*(*F*^2^) = 0.124	H atoms treated by a mixture of independent and constrained refinement
*S* = 1.03	*w* = 1/[σ^2^(*F*_o_^2^) + (0.0481*P*)^2^ + 1.1188*P*] where *P* = (*F*_o_^2^ + 2*F*_c_^2^)/3
9360 reflections	(Δ/σ)_max_ = 0.001
541 parameters	Δρ_max_ = 0.32 e Å^−^^3^
6 restraints	Δρ_min_ = −0.29 e Å^−^^3^

### Special details

**Table:**

Geometry. All s.u.'s (except the s.u. in the dihedral angle between two l.s. planes) are estimated using the full covariance matrix. The cell s.u.'s are taken into account individually in the estimation of s.u.'s in distances, angles and torsion angles; correlations between s.u.'s in cell parameters are only used when they are defined by crystal symmetry. An approximate (isotropic) treatment of cell s.u.'s is used for estimating s.u.'s involving l.s. planes.
Refinement. Refinement of *F*^2^ against ALL reflections. The weighted *R*-factor *wR* and goodness of fit *S* are based on *F*^2^, conventional *R*-factors *R* are based on *F*, with *F* set to zero for negative *F*^2^. The threshold expression of *F*^2^ > 2σ(*F*^2^) is used only for calculating *R*-factors(gt) *etc*. and is not relevant to the choice of reflections for refinement. *R*-factors based on *F*^2^ are statistically about twice as large as those based on *F*, and *R*- factors based on ALL data will be even larger.

### Fractional atomic coordinates and isotropic or equivalent isotropic displacement parameters (Å^2^)

**Table:**

	*x*	*y*	*z*	*U*_iso_*/*U*_eq_	
S1	0.93702 (5)	0.53450 (3)	0.58907 (3)	0.01735 (13)	
S2	0.17284 (5)	0.50989 (3)	0.85324 (3)	0.01659 (13)	
O1	0.40819 (13)	0.53199 (8)	0.72332 (7)	0.0199 (3)	
O2	0.67598 (14)	0.63628 (8)	0.47184 (7)	0.0210 (4)	
O3	0.70635 (14)	0.55121 (9)	0.71894 (7)	0.0264 (4)	
O4	0.43449 (14)	0.60561 (8)	0.97848 (7)	0.0223 (4)	
N1	0.21714 (17)	0.52663 (11)	0.67687 (9)	0.0220 (4)	
H11	0.199 (2)	0.5186 (13)	0.7190 (7)	0.026*	
H12	0.1584 (16)	0.5335 (13)	0.6434 (9)	0.026*	
N2	0.73227 (16)	0.60455 (9)	0.58448 (8)	0.0142 (4)	
H2	0.7165 (19)	0.5772 (10)	0.6189 (8)	0.017*	
N3	0.89631 (16)	0.67559 (9)	0.56959 (8)	0.0158 (4)	
N4	0.89252 (18)	0.52334 (12)	0.76290 (9)	0.0269 (5)	
H41	0.913 (2)	0.5191 (13)	0.7206 (7)	0.032*	
H42	0.9465 (17)	0.5154 (13)	0.7987 (9)	0.032*	
N5	0.38079 (16)	0.57719 (10)	0.86445 (8)	0.0153 (4)	
H5	0.398 (2)	0.5524 (10)	0.8289 (8)	0.018*	
N6	0.21864 (15)	0.64993 (9)	0.87808 (8)	0.0145 (4)	
C1	0.33211 (19)	0.53734 (11)	0.67209 (10)	0.0152 (4)	
C2	0.36657 (19)	0.55656 (11)	0.60158 (10)	0.0136 (4)	
C3	0.2863 (2)	0.55708 (11)	0.54047 (10)	0.0162 (5)	
H3A	0.2053	0.5450	0.5422	0.019*	
C4	0.3243 (2)	0.57522 (11)	0.47723 (10)	0.0177 (5)	
H4	0.2693	0.5760	0.4358	0.021*	
C5	0.4421 (2)	0.59218 (11)	0.47450 (10)	0.0168 (5)	
H5A	0.4678	0.6040	0.4310	0.020*	
C6	0.52415 (19)	0.59210 (11)	0.53509 (10)	0.0147 (4)	
C7	0.48522 (19)	0.57448 (11)	0.59866 (10)	0.0143 (4)	
H7	0.5399	0.5747	0.6402	0.017*	
C8	0.64912 (19)	0.61286 (11)	0.52683 (10)	0.0145 (4)	
C9	0.85511 (19)	0.61048 (11)	0.57992 (10)	0.0140 (4)	
C10	1.02249 (19)	0.68788 (12)	0.56284 (11)	0.0183 (5)	
H10A	1.0702	0.6479	0.5857	0.022*	
H10B	1.0488	0.7329	0.5875	0.022*	
C11	1.04565 (19)	0.69331 (11)	0.48772 (11)	0.0180 (5)	
C12	1.1260 (2)	0.74421 (12)	0.46958 (12)	0.0231 (5)	
H12A	1.1638	0.7757	0.5042	0.028*	
C13	1.1516 (2)	0.74958 (13)	0.40163 (12)	0.0285 (6)	
H13	1.2069	0.7845	0.3899	0.034*	
C14	1.0967 (2)	0.70406 (13)	0.35064 (12)	0.0272 (6)	
H14	1.1141	0.7077	0.3040	0.033*	
C15	1.0163 (2)	0.65326 (12)	0.36807 (11)	0.0236 (5)	
H15	0.9787	0.6219	0.3332	0.028*	
C16	0.9903 (2)	0.64788 (12)	0.43625 (11)	0.0204 (5)	
H16	0.9346	0.6131	0.4478	0.025*	
C17	0.8272 (2)	0.74278 (11)	0.57196 (10)	0.0170 (5)	
H17A	0.7414	0.7320	0.5612	0.020*	
H17B	0.8491	0.7765	0.5361	0.020*	
C18	0.85085 (19)	0.77774 (12)	0.64324 (10)	0.0175 (5)	
C19	0.8366 (2)	0.73848 (13)	0.70278 (11)	0.0248 (5)	
H19	0.8149	0.6892	0.6988	0.030*	
C20	0.8538 (2)	0.77069 (15)	0.76777 (11)	0.0320 (6)	
H20	0.8445	0.7434	0.8083	0.038*	
C21	0.8845 (2)	0.84245 (16)	0.77384 (12)	0.0352 (7)	
H21	0.8949	0.8647	0.8184	0.042*	
C22	0.9000 (2)	0.88180 (14)	0.71529 (12)	0.0302 (6)	
H22	0.9214	0.9311	0.7195	0.036*	
C23	0.8841 (2)	0.84918 (12)	0.64974 (11)	0.0233 (5)	
H23	0.8962	0.8761	0.6095	0.028*	
C24	0.7812 (2)	0.54205 (12)	0.76991 (11)	0.0177 (5)	
C25	0.74779 (19)	0.55129 (11)	0.84237 (10)	0.0154 (4)	
C26	0.82982 (19)	0.54898 (11)	0.90265 (10)	0.0161 (5)	
H26	0.9118	0.5418	0.8992	0.019*	
C27	0.7918 (2)	0.55711 (11)	0.96753 (11)	0.0183 (5)	
H27	0.8480	0.5563	1.0084	0.022*	
C28	0.6731 (2)	0.56643 (11)	0.97299 (11)	0.0176 (5)	
H28	0.6475	0.5706	1.0177	0.021*	
C29	0.58962 (19)	0.56988 (11)	0.91318 (10)	0.0153 (4)	
C30	0.62837 (19)	0.56305 (11)	0.84800 (10)	0.0152 (4)	
H30	0.5728	0.5665	0.8070	0.018*	
C31	0.46309 (19)	0.58536 (11)	0.92285 (10)	0.0160 (5)	
C32	0.25740 (19)	0.58442 (11)	0.86691 (10)	0.0149 (4)	
C33	0.09242 (19)	0.66406 (12)	0.88222 (10)	0.0168 (5)	
H33A	0.0444	0.6245	0.8588	0.020*	
H33B	0.0691	0.7091	0.8568	0.020*	
C34	0.06454 (19)	0.67085 (11)	0.95643 (10)	0.0157 (5)	
C35	−0.0182 (2)	0.72124 (12)	0.97195 (11)	0.0204 (5)	
H35	−0.0549	0.7518	0.9362	0.024*	
C36	−0.0483 (2)	0.72755 (13)	1.03892 (11)	0.0244 (5)	
H36	−0.1056	0.7621	1.0487	0.029*	
C37	0.0052 (2)	0.68354 (12)	1.09175 (11)	0.0231 (5)	
H37	−0.0149	0.6879	1.1378	0.028*	
C38	0.0882 (2)	0.63322 (12)	1.07676 (11)	0.0208 (5)	
H38	0.1247	0.6028	1.1127	0.025*	
C39	0.1185 (2)	0.62688 (11)	1.00976 (11)	0.0184 (5)	
H39	0.1761	0.5925	1.0001	0.022*	
C40	0.2908 (2)	0.71634 (11)	0.87810 (10)	0.0160 (5)	
H40A	0.3763	0.7043	0.8885	0.019*	
H40B	0.2700	0.7495	0.9147	0.019*	
C41	0.26756 (19)	0.75252 (11)	0.80750 (10)	0.0160 (5)	
C42	0.2201 (2)	0.82148 (12)	0.80117 (11)	0.0196 (5)	
H42A	0.2005	0.8459	0.8413	0.023*	
C43	0.2012 (2)	0.85472 (12)	0.73647 (11)	0.0231 (5)	
H43	0.1696	0.9020	0.7325	0.028*	
C44	0.2286 (2)	0.81875 (13)	0.67745 (11)	0.0250 (5)	
H44	0.2161	0.8415	0.6331	0.030*	
C45	0.2740 (2)	0.74981 (13)	0.68339 (11)	0.0240 (5)	
H45	0.2915	0.7249	0.6430	0.029*	
C46	0.2941 (2)	0.71683 (12)	0.74821 (11)	0.0205 (5)	
H46	0.3261	0.6696	0.7521	0.025*	

### Atomic displacement parameters (Å^2^)

**Table:**

	*U*^11^	*U*^22^	*U*^33^	*U*^12^	*U*^13^	*U*^23^
S1	0.0137 (3)	0.0162 (3)	0.0218 (3)	0.0011 (2)	0.0011 (2)	−0.0003 (2)
S2	0.0163 (3)	0.0158 (3)	0.0177 (3)	−0.0012 (2)	0.0022 (2)	0.0004 (2)
O1	0.0151 (9)	0.0305 (9)	0.0134 (7)	−0.0009 (7)	−0.0006 (6)	0.0003 (6)
O2	0.0203 (9)	0.0282 (9)	0.0144 (7)	−0.0040 (7)	0.0017 (6)	0.0044 (6)
O3	0.0155 (9)	0.0469 (11)	0.0165 (8)	0.0048 (7)	0.0010 (6)	0.0080 (7)
O4	0.0217 (10)	0.0307 (9)	0.0149 (7)	0.0043 (7)	0.0034 (6)	−0.0008 (7)
N1	0.0131 (11)	0.0370 (12)	0.0154 (9)	−0.0026 (9)	0.0000 (8)	0.0048 (9)
N2	0.0117 (10)	0.0179 (10)	0.0131 (8)	−0.0008 (7)	0.0023 (7)	0.0038 (7)
N3	0.0139 (10)	0.0169 (9)	0.0168 (9)	−0.0014 (7)	0.0029 (7)	0.0000 (7)
N4	0.0157 (12)	0.0496 (14)	0.0150 (9)	0.0084 (9)	0.0011 (8)	0.0006 (9)
N5	0.0120 (10)	0.0205 (10)	0.0141 (9)	0.0012 (7)	0.0040 (7)	−0.0029 (7)
N6	0.0128 (10)	0.0175 (10)	0.0138 (8)	0.0011 (7)	0.0033 (7)	0.0012 (7)
C1	0.0152 (12)	0.0139 (11)	0.0167 (10)	−0.0001 (8)	0.0028 (8)	−0.0032 (8)
C2	0.0144 (12)	0.0095 (10)	0.0165 (10)	0.0001 (8)	0.0012 (8)	−0.0018 (8)
C3	0.0133 (12)	0.0142 (11)	0.0210 (11)	−0.0016 (8)	0.0012 (8)	−0.0020 (9)
C4	0.0185 (13)	0.0176 (11)	0.0156 (10)	−0.0004 (9)	−0.0028 (9)	−0.0007 (9)
C5	0.0197 (13)	0.0164 (11)	0.0143 (10)	−0.0009 (9)	0.0020 (8)	0.0012 (8)
C6	0.0154 (12)	0.0105 (10)	0.0179 (10)	0.0005 (8)	0.0014 (8)	0.0003 (8)
C7	0.0147 (12)	0.0126 (10)	0.0150 (10)	0.0006 (8)	−0.0002 (8)	−0.0015 (8)
C8	0.0164 (12)	0.0124 (11)	0.0148 (10)	0.0006 (8)	0.0019 (8)	−0.0001 (8)
C9	0.0134 (12)	0.0166 (11)	0.0116 (9)	−0.0015 (8)	0.0005 (8)	−0.0003 (8)
C10	0.0135 (12)	0.0174 (11)	0.0237 (11)	−0.0042 (9)	0.0019 (9)	−0.0011 (9)
C11	0.0146 (12)	0.0158 (11)	0.0241 (11)	0.0028 (9)	0.0048 (9)	0.0021 (9)
C12	0.0225 (14)	0.0170 (12)	0.0309 (12)	−0.0008 (9)	0.0076 (10)	−0.0005 (10)
C13	0.0252 (15)	0.0248 (13)	0.0387 (14)	0.0003 (10)	0.0157 (11)	0.0062 (11)
C14	0.0268 (15)	0.0299 (14)	0.0271 (12)	0.0065 (11)	0.0123 (10)	0.0065 (11)
C15	0.0217 (14)	0.0243 (13)	0.0249 (12)	0.0046 (10)	0.0040 (10)	−0.0016 (10)
C16	0.0199 (13)	0.0163 (11)	0.0257 (12)	0.0000 (9)	0.0052 (9)	0.0034 (9)
C17	0.0186 (13)	0.0161 (11)	0.0159 (10)	0.0009 (9)	0.0003 (9)	0.0010 (8)
C18	0.0147 (13)	0.0209 (12)	0.0166 (10)	0.0034 (9)	0.0014 (8)	−0.0007 (9)
C19	0.0238 (14)	0.0306 (14)	0.0203 (11)	0.0011 (10)	0.0042 (9)	0.0017 (10)
C20	0.0287 (16)	0.0520 (18)	0.0155 (11)	0.0047 (12)	0.0040 (10)	0.0018 (11)
C21	0.0278 (16)	0.0552 (19)	0.0211 (12)	0.0120 (13)	−0.0029 (10)	−0.0155 (12)
C22	0.0285 (16)	0.0266 (14)	0.0323 (13)	0.0067 (11)	−0.0081 (11)	−0.0114 (11)
C23	0.0236 (14)	0.0214 (12)	0.0234 (11)	0.0037 (10)	−0.0028 (10)	−0.0014 (10)
C24	0.0148 (13)	0.0194 (12)	0.0191 (10)	−0.0003 (9)	0.0024 (9)	0.0032 (9)
C25	0.0151 (12)	0.0134 (11)	0.0176 (10)	0.0000 (8)	0.0021 (8)	0.0027 (8)
C26	0.0123 (12)	0.0155 (11)	0.0201 (10)	−0.0009 (8)	−0.0002 (8)	0.0038 (9)
C27	0.0172 (13)	0.0183 (11)	0.0177 (10)	−0.0009 (9)	−0.0040 (9)	0.0017 (9)
C28	0.0196 (13)	0.0171 (11)	0.0159 (10)	−0.0007 (9)	0.0021 (9)	0.0019 (9)
C29	0.0164 (12)	0.0123 (11)	0.0170 (10)	−0.0006 (8)	0.0014 (8)	0.0007 (8)
C30	0.0153 (12)	0.0145 (11)	0.0153 (10)	0.0003 (8)	−0.0005 (8)	0.0019 (8)
C31	0.0166 (12)	0.0147 (11)	0.0166 (10)	0.0005 (8)	0.0015 (9)	0.0025 (8)
C32	0.0149 (12)	0.0197 (11)	0.0101 (9)	0.0009 (9)	0.0010 (8)	0.0015 (8)
C33	0.0136 (12)	0.0178 (11)	0.0190 (10)	0.0027 (9)	0.0021 (8)	0.0019 (9)
C34	0.0145 (12)	0.0136 (11)	0.0195 (10)	−0.0041 (8)	0.0044 (9)	−0.0010 (9)
C35	0.0204 (14)	0.0180 (12)	0.0231 (11)	0.0036 (9)	0.0043 (9)	0.0016 (9)
C36	0.0220 (14)	0.0253 (13)	0.0276 (12)	0.0042 (10)	0.0092 (10)	−0.0030 (10)
C37	0.0255 (14)	0.0253 (13)	0.0203 (11)	−0.0021 (10)	0.0095 (10)	−0.0027 (10)
C38	0.0220 (14)	0.0210 (12)	0.0191 (11)	−0.0017 (9)	0.0012 (9)	0.0034 (9)
C39	0.0169 (13)	0.0161 (11)	0.0228 (11)	0.0018 (9)	0.0048 (9)	0.0009 (9)
C40	0.0190 (13)	0.0129 (11)	0.0160 (10)	−0.0017 (8)	0.0027 (8)	−0.0014 (8)
C41	0.0144 (12)	0.0167 (11)	0.0171 (10)	−0.0038 (8)	0.0031 (8)	−0.0001 (8)
C42	0.0206 (13)	0.0191 (12)	0.0192 (11)	−0.0007 (9)	0.0038 (9)	−0.0011 (9)
C43	0.0241 (14)	0.0194 (12)	0.0255 (12)	0.0000 (10)	0.0024 (10)	0.0036 (10)
C44	0.0276 (15)	0.0286 (14)	0.0184 (11)	−0.0058 (10)	0.0016 (10)	0.0064 (10)
C45	0.0306 (15)	0.0241 (13)	0.0189 (11)	−0.0045 (10)	0.0093 (10)	−0.0034 (10)
C46	0.0233 (14)	0.0172 (12)	0.0216 (11)	−0.0017 (9)	0.0053 (9)	−0.0004 (9)

### Geometric parameters (Å, º)

**Table:**

S1—C9	1.689 (2)	C17—H17B	0.9900
S2—C32	1.688 (2)	C18—C23	1.384 (3)
O1—C1	1.232 (2)	C18—C19	1.390 (3)
O2—C8	1.223 (2)	C19—C20	1.383 (3)
O3—C24	1.228 (2)	C19—H19	0.9500
O4—C31	1.222 (2)	C20—C21	1.381 (4)
N1—C1	1.334 (3)	C20—H20	0.9500
N1—H11	0.877 (9)	C21—C22	1.378 (4)
N1—H12	0.877 (9)	C21—H21	0.9500
N2—C8	1.374 (3)	C22—C23	1.396 (3)
N2—C9	1.412 (3)	C22—H22	0.9500
N2—H2	0.874 (9)	C23—H23	0.9500
N3—C9	1.323 (3)	C24—C25	1.506 (3)
N3—C10	1.472 (3)	C25—C30	1.390 (3)
N3—C17	1.480 (3)	C25—C26	1.396 (3)
N4—C24	1.333 (3)	C26—C27	1.385 (3)
N4—H41	0.882 (10)	C26—H26	0.9500
N4—H42	0.876 (10)	C27—C28	1.375 (3)
N5—C31	1.379 (3)	C27—H27	0.9500
N5—C32	1.413 (3)	C28—C29	1.400 (3)
N5—H5	0.872 (9)	C28—H28	0.9500
N6—C32	1.323 (3)	C29—C30	1.392 (3)
N6—C33	1.468 (3)	C29—C31	1.499 (3)
N6—C40	1.483 (3)	C30—H30	0.9500
C1—C2	1.508 (3)	C33—C34	1.513 (3)
C2—C7	1.395 (3)	C33—H33A	0.9900
C2—C3	1.397 (3)	C33—H33B	0.9900
C3—C4	1.389 (3)	C34—C35	1.387 (3)
C3—H3A	0.9500	C34—C39	1.395 (3)
C4—C5	1.380 (3)	C35—C36	1.385 (3)
C4—H4	0.9500	C35—H35	0.9500
C5—C6	1.400 (3)	C36—C37	1.387 (3)
C5—H5A	0.9500	C36—H36	0.9500
C6—C7	1.396 (3)	C37—C38	1.384 (3)
C6—C8	1.498 (3)	C37—H37	0.9500
C7—H7	0.9500	C38—C39	1.386 (3)
C10—C11	1.511 (3)	C38—H38	0.9500
C10—H10A	0.9900	C39—H39	0.9500
C10—H10B	0.9900	C40—C41	1.514 (3)
C11—C12	1.390 (3)	C40—H40A	0.9900
C11—C16	1.393 (3)	C40—H40B	0.9900
C12—C13	1.384 (3)	C41—C46	1.390 (3)
C12—H12A	0.9500	C41—C42	1.391 (3)
C13—C14	1.386 (3)	C42—C43	1.387 (3)
C13—H13	0.9500	C42—H42A	0.9500
C14—C15	1.385 (3)	C43—C44	1.391 (3)
C14—H14	0.9500	C43—H43	0.9500
C15—C16	1.390 (3)	C44—C45	1.382 (3)
C15—H15	0.9500	C44—H44	0.9500
C16—H16	0.9500	C45—C46	1.388 (3)
C17—C18	1.516 (3)	C45—H45	0.9500
C17—H17A	0.9900	C46—H46	0.9500
			
C1—N1—H11	115.9 (16)	C22—C21—C20	120.1 (2)
C1—N1—H12	125.8 (16)	C22—C21—H21	120.0
H11—N1—H12	118 (2)	C20—C21—H21	120.0
C8—N2—C9	121.46 (17)	C21—C22—C23	120.0 (2)
C8—N2—H2	119.8 (15)	C21—C22—H22	120.0
C9—N2—H2	113.1 (15)	C23—C22—H22	120.0
C9—N3—C10	121.47 (18)	C18—C23—C22	120.2 (2)
C9—N3—C17	124.78 (19)	C18—C23—H23	119.9
C10—N3—C17	113.37 (17)	C22—C23—H23	119.9
C24—N4—H41	118.9 (16)	O3—C24—N4	121.5 (2)
C24—N4—H42	122.7 (17)	O3—C24—C25	120.0 (2)
H41—N4—H42	118 (2)	N4—C24—C25	118.53 (18)
C31—N5—C32	122.19 (17)	C30—C25—C26	119.49 (19)
C31—N5—H5	120.2 (15)	C30—C25—C24	116.97 (17)
C32—N5—H5	113.9 (15)	C26—C25—C24	123.5 (2)
C32—N6—C33	121.38 (18)	C27—C26—C25	120.1 (2)
C32—N6—C40	124.96 (19)	C27—C26—H26	120.0
C33—N6—C40	113.16 (17)	C25—C26—H26	120.0
O1—C1—N1	121.57 (19)	C28—C27—C26	120.23 (19)
O1—C1—C2	120.66 (19)	C28—C27—H27	119.9
N1—C1—C2	117.78 (17)	C26—C27—H27	119.9
C7—C2—C3	119.54 (19)	C27—C28—C29	120.6 (2)
C7—C2—C1	117.11 (17)	C27—C28—H28	119.7
C3—C2—C1	123.35 (19)	C29—C28—H28	119.7
C4—C3—C2	120.2 (2)	C30—C29—C28	119.0 (2)
C4—C3—H3A	119.9	C30—C29—C31	123.23 (18)
C2—C3—H3A	119.9	C28—C29—C31	117.65 (18)
C5—C4—C3	119.99 (19)	C25—C30—C29	120.54 (18)
C5—C4—H4	120.0	C25—C30—H30	119.7
C3—C4—H4	120.0	C29—C30—H30	119.7
C4—C5—C6	120.77 (19)	O4—C31—N5	121.8 (2)
C4—C5—H5A	119.6	O4—C31—C29	122.33 (18)
C6—C5—H5A	119.6	N5—C31—C29	115.88 (18)
C7—C6—C5	119.0 (2)	N6—C32—N5	116.38 (19)
C7—C6—C8	124.33 (18)	N6—C32—S2	126.06 (17)
C5—C6—C8	116.63 (18)	N5—C32—S2	117.54 (15)
C6—C7—C2	120.43 (18)	N6—C33—C34	112.92 (16)
C6—C7—H7	119.8	N6—C33—H33A	109.0
C2—C7—H7	119.8	C34—C33—H33A	109.0
O2—C8—N2	121.6 (2)	N6—C33—H33B	109.0
O2—C8—C6	121.91 (18)	C34—C33—H33B	109.0
N2—C8—C6	116.50 (17)	H33A—C33—H33B	107.8
N3—C9—N2	116.84 (18)	C35—C34—C39	118.8 (2)
N3—C9—S1	125.61 (17)	C35—C34—C33	119.37 (18)
N2—C9—S1	117.54 (15)	C39—C34—C33	121.78 (19)
N3—C10—C11	112.66 (17)	C36—C35—C34	120.9 (2)
N3—C10—H10A	109.1	C36—C35—H35	119.6
C11—C10—H10A	109.1	C34—C35—H35	119.6
N3—C10—H10B	109.1	C35—C36—C37	120.1 (2)
C11—C10—H10B	109.1	C35—C36—H36	120.0
H10A—C10—H10B	107.8	C37—C36—H36	120.0
C12—C11—C16	119.0 (2)	C38—C37—C36	119.4 (2)
C12—C11—C10	119.17 (19)	C38—C37—H37	120.3
C16—C11—C10	121.9 (2)	C36—C37—H37	120.3
C13—C12—C11	120.8 (2)	C37—C38—C39	120.6 (2)
C13—C12—H12A	119.6	C37—C38—H38	119.7
C11—C12—H12A	119.6	C39—C38—H38	119.7
C12—C13—C14	120.1 (2)	C38—C39—C34	120.2 (2)
C12—C13—H13	120.0	C38—C39—H39	119.9
C14—C13—H13	120.0	C34—C39—H39	119.9
C13—C14—C15	119.7 (2)	N6—C40—C41	109.71 (16)
C13—C14—H14	120.2	N6—C40—H40A	109.7
C15—C14—H14	120.2	C41—C40—H40A	109.7
C14—C15—C16	120.4 (2)	N6—C40—H40B	109.7
C14—C15—H15	119.8	C41—C40—H40B	109.7
C16—C15—H15	119.8	H40A—C40—H40B	108.2
C15—C16—C11	120.2 (2)	C46—C41—C42	119.35 (19)
C15—C16—H16	119.9	C46—C41—C40	119.95 (19)
C11—C16—H16	119.9	C42—C41—C40	120.70 (19)
N3—C17—C18	110.93 (16)	C43—C42—C41	120.3 (2)
N3—C17—H17A	109.5	C43—C42—H42A	119.9
C18—C17—H17A	109.5	C41—C42—H42A	119.9
N3—C17—H17B	109.5	C42—C43—C44	120.0 (2)
C18—C17—H17B	109.5	C42—C43—H43	120.0
H17A—C17—H17B	108.0	C44—C43—H43	120.0
C23—C18—C19	119.3 (2)	C45—C44—C43	119.9 (2)
C23—C18—C17	120.64 (19)	C45—C44—H44	120.1
C19—C18—C17	120.1 (2)	C43—C44—H44	120.1
C20—C19—C18	120.4 (2)	C44—C45—C46	120.2 (2)
C20—C19—H19	119.8	C44—C45—H45	119.9
C18—C19—H19	119.8	C46—C45—H45	119.9
C21—C20—C19	120.1 (2)	C45—C46—C41	120.3 (2)
C21—C20—H20	120.0	C45—C46—H46	119.8
C19—C20—H20	120.0	C41—C46—H46	119.8
			
O1—C1—C2—C7	−6.0 (3)	O3—C24—C25—C30	6.3 (3)
N1—C1—C2—C7	173.88 (19)	N4—C24—C25—C30	−173.2 (2)
O1—C1—C2—C3	174.0 (2)	O3—C24—C25—C26	−173.3 (2)
N1—C1—C2—C3	−6.2 (3)	N4—C24—C25—C26	7.2 (3)
C7—C2—C3—C4	0.1 (3)	C30—C25—C26—C27	1.1 (3)
C1—C2—C3—C4	−179.87 (19)	C24—C25—C26—C27	−179.22 (19)
C2—C3—C4—C5	0.6 (3)	C25—C26—C27—C28	1.0 (3)
C3—C4—C5—C6	−0.7 (3)	C26—C27—C28—C29	−1.8 (3)
C4—C5—C6—C7	0.1 (3)	C27—C28—C29—C30	0.6 (3)
C4—C5—C6—C8	−178.66 (19)	C27—C28—C29—C31	−175.72 (19)
C5—C6—C7—C2	0.6 (3)	C26—C25—C30—C29	−2.4 (3)
C8—C6—C7—C2	179.24 (19)	C24—C25—C30—C29	177.97 (19)
C3—C2—C7—C6	−0.7 (3)	C28—C29—C30—C25	1.5 (3)
C1—C2—C7—C6	179.27 (18)	C31—C29—C30—C25	177.62 (19)
C9—N2—C8—O2	−11.3 (3)	C32—N5—C31—O4	−6.7 (3)
C9—N2—C8—C6	169.02 (18)	C32—N5—C31—C29	174.95 (18)
C7—C6—C8—O2	−170.9 (2)	C30—C29—C31—O4	−164.7 (2)
C5—C6—C8—O2	7.8 (3)	C28—C29—C31—O4	11.5 (3)
C7—C6—C8—N2	8.8 (3)	C30—C29—C31—N5	13.7 (3)
C5—C6—C8—N2	−172.49 (18)	C28—C29—C31—N5	−170.16 (19)
C10—N3—C9—N2	−178.97 (16)	C33—N6—C32—N5	−179.56 (16)
C17—N3—C9—N2	8.6 (3)	C40—N6—C32—N5	9.1 (3)
C10—N3—C9—S1	1.9 (3)	C33—N6—C32—S2	2.2 (3)
C17—N3—C9—S1	−170.59 (15)	C40—N6—C32—S2	−169.12 (14)
C8—N2—C9—N3	71.5 (2)	C31—N5—C32—N6	69.4 (2)
C8—N2—C9—S1	−109.29 (19)	C31—N5—C32—S2	−112.29 (19)
C9—N3—C10—C11	98.2 (2)	C32—N6—C33—C34	100.5 (2)
C17—N3—C10—C11	−88.5 (2)	C40—N6—C33—C34	−87.2 (2)
N3—C10—C11—C12	140.0 (2)	N6—C33—C34—C35	142.2 (2)
N3—C10—C11—C16	−41.2 (3)	N6—C33—C34—C39	−38.9 (3)
C16—C11—C12—C13	−0.5 (3)	C39—C34—C35—C36	−0.7 (3)
C10—C11—C12—C13	178.4 (2)	C33—C34—C35—C36	178.2 (2)
C11—C12—C13—C14	0.2 (4)	C34—C35—C36—C37	0.5 (4)
C12—C13—C14—C15	0.0 (4)	C35—C36—C37—C38	−0.3 (4)
C13—C14—C15—C16	0.2 (4)	C36—C37—C38—C39	0.4 (3)
C14—C15—C16—C11	−0.4 (3)	C37—C38—C39—C34	−0.6 (3)
C12—C11—C16—C15	0.6 (3)	C35—C34—C39—C38	0.8 (3)
C10—C11—C16—C15	−178.3 (2)	C33—C34—C39—C38	−178.1 (2)
C9—N3—C17—C18	95.8 (2)	C32—N6—C40—C41	96.9 (2)
C10—N3—C17—C18	−77.2 (2)	C33—N6—C40—C41	−75.1 (2)
N3—C17—C18—C23	127.9 (2)	N6—C40—C41—C46	−61.5 (3)
N3—C17—C18—C19	−53.6 (3)	N6—C40—C41—C42	118.7 (2)
C23—C18—C19—C20	0.9 (4)	C46—C41—C42—C43	−1.0 (3)
C17—C18—C19—C20	−177.6 (2)	C40—C41—C42—C43	178.8 (2)
C18—C19—C20—C21	0.5 (4)	C41—C42—C43—C44	0.7 (3)
C19—C20—C21—C22	−1.1 (4)	C42—C43—C44—C45	0.3 (4)
C20—C21—C22—C23	0.3 (4)	C43—C44—C45—C46	−1.0 (4)
C19—C18—C23—C22	−1.7 (3)	C44—C45—C46—C41	0.7 (4)
C17—C18—C23—C22	176.8 (2)	C42—C41—C46—C45	0.3 (3)
C21—C22—C23—C18	1.1 (4)	C40—C41—C46—C45	−179.4 (2)

### Hydrogen-bond geometry (Å, º)

Cg1–Cg3 are the centroids of the C25–C30, C34–C39 and C11–C16 benzene rings, respectively.

**Table:**

*D*—H···*A*	*D*—H	H···*A*	*D*···*A*	*D*—H···*A*
N1—H12···S1^i^	0.88 (1)	2.60 (1)	3.4103 (19)	155 (2)
N1—H11···S2	0.88 (1)	2.65 (1)	3.5220 (19)	170 (2)
N2—H2···O3	0.87 (1)	2.01 (1)	2.832 (2)	156 (2)
N4—H41···S1	0.88 (1)	2.61 (1)	3.4666 (19)	165 (2)
N4—H42···S2^ii^	0.88 (1)	2.65 (2)	3.437 (2)	150 (2)
N5—H5···O1	0.87 (1)	2.09 (1)	2.909 (2)	156 (2)
C13—H13···*Cg*1^iii^	0.95	2.92	3.614 (3)	130
C17—H17*A*···*Cg*2^iii^	0.99	2.90	3.644 (2)	132
C40—H40*A*···*Cg*3^iv^	0.99	2.86	3.549 (2)	128

Symmetry codes: (i) *x*−1, *y*, *z*; (ii) *x*+1, *y*, *z*; (iii) *x*+1/2, −*y*+3/2, *z*−1/2; (iv) *x*−1/2, −*y*+3/2, *z*+1/2.
